# Editorial: Bridging computation, biophysics, medicine, and engineering in neural circuits

**DOI:** 10.3389/fncir.2026.1776224

**Published:** 2026-01-21

**Authors:** Haruyuki Kamiya, Hideaki Yamamoto, Jordi Soriano, Shigeo Sato, Toshiaki Omori

**Affiliations:** 1 Department of Neurobiology, Graduate School of Medicine, Hokkaido University, Sapporo, Japan; 2 Research Institute of Electrical Communication, Tohoku University, Sendai, Japan; 3 Departament de Física de la Matèria Condensada, Universitat de Barcelona, Barcelona, Spain; 4 Department of Electrical and Electronic Engineering, Graduate School of Engineering, Kobe University, Kobe, Japan

**Keywords:** biophysics, complex networks, computational neuroscience, information processing, neural circuits, neuromorphic hardware, neurophysiology, signal processing

Neural circuits in the brain are highly energy-efficient biological hardware enabling ultra-fast parallel computation that integrates sensory inputs, motor outputs, and internal memory processes. However, the mechanisms underlying energy-efficient computation in biological neural circuits need to be unraveled with a wide range of multidisciplinary approaches. This Research Topic aims to facilitate mutual understanding between the *in silico, in vitro*, and *in vivo* neuroscientists with diverse backgrounds. To this end, a wide variety of cutting-edge topics are collected from research in computational neuroscience, biophysics, neurophysiology, material sciences, neuromorphic hardware, bioengineering, and medicine. A unifying theme across these works is the investigation of how structured dynamics and learning rules enable robust information processing, cognition, and recovery from damage in biological neural systems, as well as how these principles can guide the development of computational and neuroengineering models.

Several studies in this Research Topic focus on recurrent network dynamics as a foundation for cortical information processing. Yonemura and Katori extended reservoir computing-based cortical models to account for multi-sensory integration. By combining predictive coding with reliability-weighted error feedback, their model allows a recurrent reservoir to extract temporal context while dynamically adjusting the influence of each sensory modality depending on its noise level. *In silico* simulations demonstrate that recurrent dynamics can flexibly integrate information across modalities, supporting robust speech recognition. These findings highlight reservoir computing as a biologically plausible and powerful approach for modeling multi-sensory integration in the cortex, where noise and uncertainty are intrinsic features.

At the level of synaptic learning rules, Orima et al. investigated the spatiotemporal learning rule (STLR), which has been proposed to reproduce hippocampal synaptic plasticity. While prior studies mainly evaluated network outputs, Orima et al. emphasized the importance of analyzing synaptic weights themselves to understand the underlying learning dynamics. By mapping synaptic weights into a distance space and applying multiple analytical methods including multidimensional scaling, fractal dimension estimation, and iterated function system modeling, they showed that STLR organizes synaptic weights into a fractal-like structure. This fractal coding suggests that synaptic plasticity rules may generate highly structured, self-similar weight distributions, offering a novel perspective on how complex memory representations can emerge from local learning rules.

Oscillatory dynamics and their functional roles are another major focus of this Research Topic. Masoliver et al. examined hippocampal theta rhythms, in which interneurons are phase-locked while pyramidal neurons exhibit phase precession. Noting the coexistence of synchrony and asynchrony, the authors proposed that this phenomenon resembles chimera states. Using Kuramoto oscillator networks that support chimera dynamics, they modeled the experimentally observed frequency relationship between theta oscillations (~8 Hz) and phase-precession pyramidal neurons (~9 Hz). Moreover, faster oscillators exhibited theta-sequence-like ordering. Importantly, spiking neural networks trained on these chimera dynamics produced subsets of neurons showing phase precession, suggesting that chimera-like dynamics may underlie key temporal coding mechanisms in the hippocampus.

Gamma oscillations, which are closely associated with cognitive functions, were addressed by Li et al., who developed a biologically plausible low-rank spiking neural network based on the voltage-dependent theta neuron model. Through macroscopic analysis, they identified network regimes ranging from stationary firing to gamma oscillations driven by structured connectivity. The model successfully reproduced phase-dependent modulation of neuronal responses in a Go-Nogo task and demonstrated that gamma oscillations can enhance and prolong signal responses. This work provides a mechanistic explanation for how oscillatory dynamics influence cognitive processing and extends low-rank spiking neural network models to capture population-level synchrony.

Predictive representations and reinforcement learning were explored by Tsurumi and Morita through actor–critic neural network models incorporating the successor representation (SR), a predictive state representation thought to be relevant to hippocampal function. By systematically varying whether the actor, the critic, or both components used SR, and comparing these configurations with Q-learning and SARSA, the authors revealed distinct functional roles of SR in different components of the learning architecture. They further showed that combining SR with one-hot state encoding yielded complementary benefits, suggesting that multiple state representations may coexist and cooperate during learning. These findings support the idea that the striatum and related circuits may employ heterogeneous representations to optimize action selection.

Network robustness and recovery after injury were addressed by Sumi et al., who modeled damage and recovery in a modular *in vitro* neuronal network. Their simulations reproduced experimental observations of reduced spontaneous collective activity following focal lesions and recovery within 24 h. By incorporating spike-timing-dependent plasticity (STDP), the model captured both the decline and restoration of global activity. The results indicate that modular organization, together with synaptic plasticity, enhances network resilience, supports functional recovery, and improves information representation during the post-injury phase.

At the single-neuron and axonal level, Kamiya investigated afterdischarge, prolonged increases in excitability following strong repetitive stimulation, using a computational model of hippocampal mossy fibers with realistic ionic conductances. The study showed that slight depolarization of distal axons combined with high-frequency stimulation can induce robust, long-lasting afterdischarges originating from distal axonal sites and propagating antidromically to the soma. Crucially, replacing inactivating potassium channels with non-inactivating ones abolished afterdischarges, demonstrating that potassium channel inactivation is essential for ectopic spike generation.

Synaptic transmission and input–output characterization were examined by Gattas et al., who applied Volterra expansion to model hippocampal CA3–CA1 synaptic transmission. They found that apical dendritic field excitatory postsynaptic potentials could be described with over 94% accuracy by a second-order model, whereas basal dendrites required higher-order non-linearities. This concise yet accurate mathematical framework offers a scalable approach for building biologically realistic network models that capture dendrite-specific processing.

Neuroengineering approaches were highlighted by Miyahara et al., who developed a microfluidics-based co-culture system of dorsal root ganglion (DRG) and spinal neurons to study spinal pain processing. Using high-density microelectrode arrays and optogenetic stimulation, they demonstrated that activating DRG neurons induced strong and prolonged synchronization in spinal networks, persisting for at least 20 min. This platform provides a valuable tool for investigating mechanisms underlying chronic and nociplastic pain.

Criticality and state-dependent dynamics were explored by Yaghoubi et al. using large-scale calcium imaging of neuronal cultures. By analyzing neuronal avalanche statistics separately during up and down states, they found scale-free behavior in both states when intrinsic time scales were considered. Down-state dynamics closely resembled those of cultures lacking up states, and standard network models failed to reproduce these observations, suggesting the need for more complex, state-dependent self-organization mechanisms.

Additional studies examined autaptic plasticity, developmental synapse properties, brain-state analysis, motor learning, and *in vitro* human brain modeling. Onda et al. showed that STDP in a minimal autaptic neuron model induces oscillatory weight changes depending on delay and stimulation frequency, potentially contributing to synchronization. Kitaoka et al. revealed positional differences in presynaptically silent synapses within single neurons, with higher proportions at distal sites. Hosaka et al. critically evaluated energy landscape analysis of resting-state fMRI, showing that many inferred state-switching features can arise from stationary linear properties. Tezuka et al. introduced a novel behavioral paradigm for studying simultaneous bimanual movements in mice, and Nishimura et al. reviewed advances in human pluripotent stem cell-derived brain organoids as platforms for modeling human brain development and disease. Finally, Onishi et al. summarized recent developments in activity-dependent gene expression tools that have emerged as powerful methods for linking neuronal activity to behavior by enabling causal analysis, thereby anticipating potential contributions to neural circuit research.

As overviewed above, the studies in this Research Topic underscore the importance of structured dynamics, plasticity, and representation in neural systems and demonstrate how integrative computational, experimental, and engineering approaches can deepen our understanding of brain function and dysfunction. It is also clear that continued progress in elucidating the dynamics and functions of neural circuits will require a sustained combination of cutting edge theoretical, engineering, and biophysical approaches.

